# End-to-end optimization of prosthetic vision

**DOI:** 10.1167/jov.22.2.20

**Published:** 2022-02-28

**Authors:** Jaap de Ruyter van Steveninck, Umut Güçlü, Richard van Wezel, Marcel van Gerven

**Affiliations:** 1 Department of Artificial Intelligence, Donders Institute for Brain, Cognition and Behaviour, Radboud University, Nijmegen, The Netherlands; 2 Department of Biophysics, Donders Institute for Brain, Cognition and Behaviour, Radboud University, Nijmegen, The Netherlands; 3 Biomedical Signal and Systems, MIRA Institute for Biomedical Technology and Technical Medicine, University of Twente, Enschede, The Netherlands

**Keywords:** prosthetic vision, deep learning, computer vision, end-to-end optimization

## Abstract

Neural prosthetics may provide a promising solution to restore visual perception in some forms of blindness. The restored prosthetic percept is rudimentary compared to normal vision and can be optimized with a variety of image preprocessing techniques to maximize relevant information transfer. Extracting the most useful features from a visual scene is a nontrivial task and optimal preprocessing choices strongly depend on the context. Despite rapid advancements in deep learning, research currently faces a difficult challenge in finding a general and automated preprocessing strategy that can be tailored to specific tasks or user requirements. In this paper, we present a novel deep learning approach that explicitly addresses this issue by optimizing the entire process of phosphene generation in an end-to-end fashion. The proposed model is based on a deep auto-encoder architecture and includes a highly adjustable simulation module of prosthetic vision. In computational validation experiments, we show that such an approach is able to automatically find a task-specific stimulation protocol. The results of these proof-of-principle experiments illustrate the potential of end-to-end optimization for prosthetic vision. The presented approach is highly modular and our approach could be extended to automated dynamic optimization of prosthetic vision for everyday tasks, given any specific constraints, accommodating individual requirements of the end-user.

## Introduction

**Figure 1.**
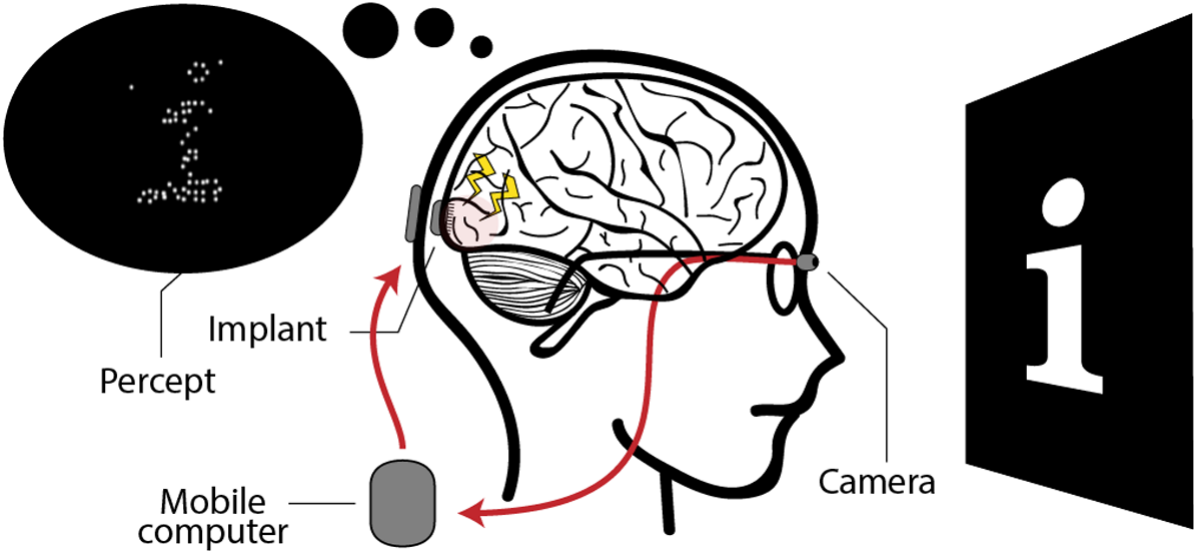
Schematic illustration of a cortical visual neuro-prosthesis. The visual environment is captured by a camera and sent to a mobile computer. Electrodes in the brain implant are selectively activated to stimulate neurons in the primary visual cortex (V1). Making use of the retinotopic organization of V1, a controlled arrangement of phosphenes can be generated to create a meaningful representation of the visual environment.

Globally, over 30 million people suffer from blindness (Stevens et al., 2013). For some forms of blindness, visual prosthetics may provide a promising solution that can restore a rudimentary form of vision (Chen, Wang Fernandez, & Roelfsema, 2020; Fernandez, 2018; Lewis et al., 2016; Lozano et al., 2020; Riazi-Esfahani, Maghami, Sodagar, Lashay, & riazi-Esfahani, 2014; Roelfsema, Denys, & Klink, 2018; Shepherd, Shivdasani, Nayagam, Williams, & Blamey, 2013). These neural interfaces can functionally replace the eye with a camera that is connected to the retina (Weiland, Liu, & Humayun, 2005; Zrenner et al., 2011), thalamus (Pezaris & Reid, 2007), or visual cortex (Beauchamp & Yoshor, 2020; Chen et al., 2020; Dobelle, Mladejovsky, & Girvin, 1974; Tehovnik & Slocum, 2013). Although, in terms of their entry point in the visual system, these types of visual prostheses may vary considerably, they share the same basic mechanism of action: through electrical stimulation of small groups of neurons, they evoke a percept of spatially localized flashes of light, called phosphenes (Brindley & Lewin, 1968; Najarpour Foroushani, Pack, & Sawan, 2018). In this paper, we focus on visual implants that reside in the primary visual cortex (V1), which are reported to have an enormous potential in future treatment of visual impairment (Beauchamp et al., 2020; Beauchamp & Yoshor, 2020; Chen et al., 2020; Lewis, Ackland, Lowery, & Rosenfeld, 2015). Due to the relatively large surface area, this implantation site allows for stimulation with many electrodes. By selective stimulation and by making use of the roughly retinotopical organization of V1, it is possible to generate a controlled arrangement of phosphenes in such a way that they may provide a meaningful representation of the visual environment (Figure 1; Chen et al., 2020).

Compared to normal vision, the percept that can be restored with visual prostheses is very rudimentary and the resolution remains relatively limited, even with relatively high numbers of electrodes. The limited amount of information that can be conveyed allows for only selective visualization of the surroundings. Therefore, an important role in the optimization of prosthetic vision will be fulfilled by image preprocessing techniques. By selective filtering of the visual environment, image preprocessing may help to maximize the usefulness and interpretability of phosphene representations. The choice of filtering is nontrivial and the definition of useful information will strongly depend on the context. Therefore, the implementation and optimization of image preprocessing techniques for prosthetic vision remain active topics of scientific investigation.

In previous work, various preprocessing techniques have been tested for a variety of applications using simulated prosthetic vision (SPV; Dagnelie et al., 2007; Parikh, Itti, Humayun, & Weiland, 2013; Srivastava, Troyk, & Dagnelie, 2009; Vergnieux, Mace, & Jouffrais, 2014). Such preprocessing techniques range from basic edge-filtering techniques for increasing wayfinding performance (Vergnieux, Macé, & Jouffrais, 2017) to more sophisticated algorithms, such as segmentation models for object recognition (Sanchez-Garcia, Martinez-Cantin, & Guerrero, 2020), or facial landmark detection algorithms for emotion recognition (Bollen, Guclu, van Wezel, van Gerven, & Gucluturk, 2019; Bollen, van Wezel, van Gerven, & Güçlütürk, 2019). The latter two examples underline the potential benefits of embracing recent breakthroughs in computer vision and deep learning models for optimization of prosthetic vision.

Despite the rapid advancements in these fields, research currently faces a difficult challenge in finding a general preprocessing strategy that can be automatically tailored to specific tasks and requirements of the user. We illustrate this with two issues: first, prosthetic engineers need to speculate or make assumptions about what visual features are crucial for the task and the ways in which these features can be transformed into a suitable stimulation protocol. Second, as a result of practical, medical, or biophysical limitations of the neural interface, one might want to tailor the stimulation parameters to additional constraints. Recent work on task-based feature learning for prosthetic vision suggests that deep learning models can be used to overcome such issues (White, Kameneva, & McCarthy, 2019).

In this paper, we present a novel approach that explicitly exploits the potential of deep learning models for automated optimization to specific tasks and constraints. We propose a deep neural network (DNN) auto-encoder architecture, that includes a highly adjustable simulation module of cortical prosthetic vision. Instead of optimizing image preprocessing as an isolated operation, our approach is designed to optimize the entire process of phosphene generation in an end-to-end fashion (Donti, Amos, & Zico Kolter, 2017). As a proof of principle, we demonstrate with computational simulations that by considering the entire pipeline as an end-to-end optimization problem, we can automatically find a stimulation protocol that optimally preserves information encoded in the phosphene representation, arriving at results that are comparable to traditional approaches. Furthermore, we show that such an approach enables tailored optimization to specific additional constraints, such as sparse electrode activation or arbitrary phosphene mappings.

## Methods

In this section, we provide an overview of the components of the proposed end-to-end deep learning architecture. Next, we describe four simulation experiments that were conducted to explore the performance of our model with various sparsity constraints, naturalistic visual contexts and realistic phosphene mappings.

### Model description

**Figure 2.**
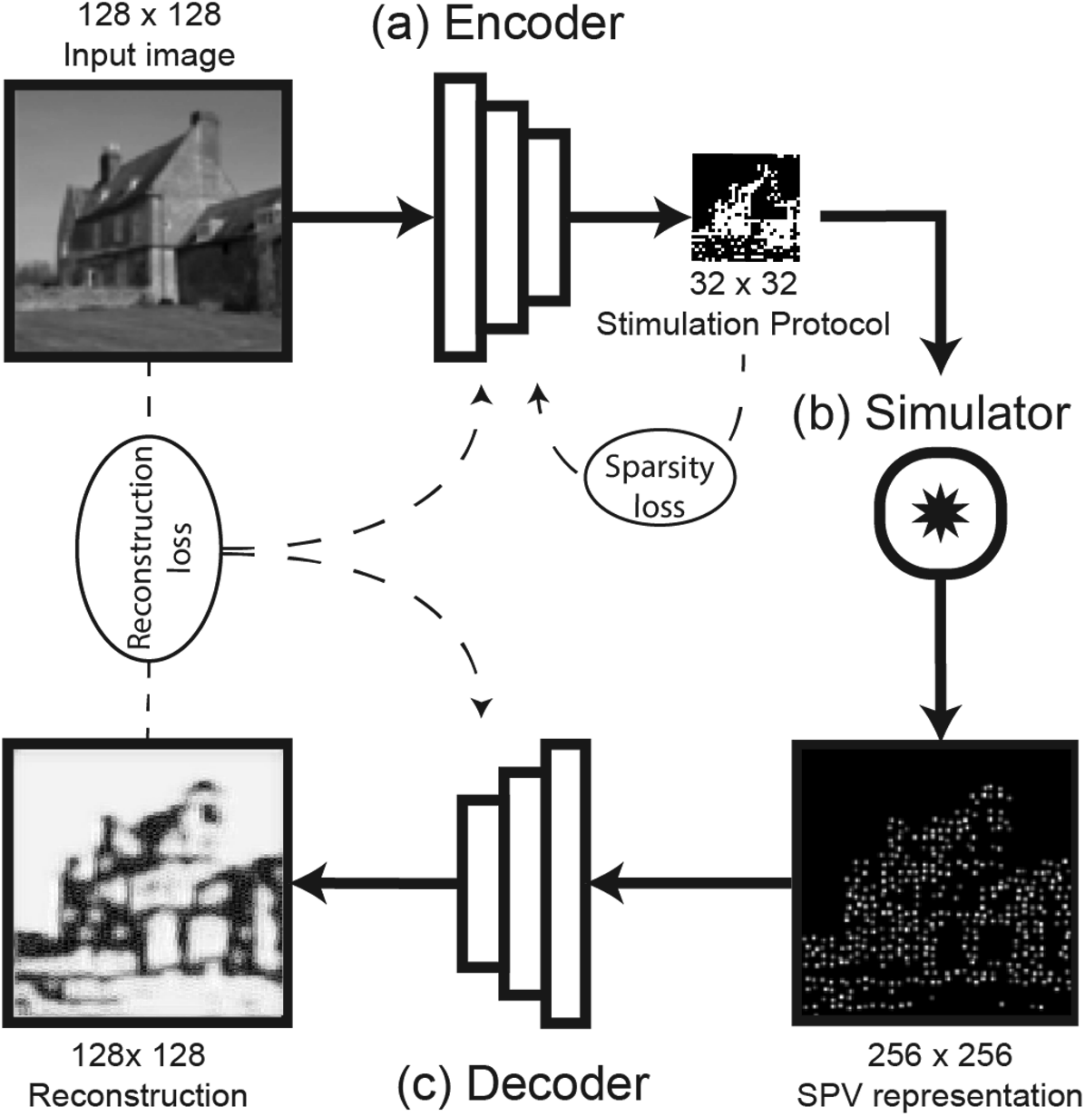
Schematic representation of the end-to-end model and its three components. (**a**) The phosphene encoder finds a stimulation protocol, given an input image. (**b**) The personalized phosphene simulator maps the stimulation vector into a simulated phosphene vision (SPV) representation. (**c**) The phosphene decoder receives a SPV-image as input and generates a reconstruction of the original image. During training, the reconstruction dissimilarity loss between the reconstructed and original image is backpropagated to the encoder and decoder models. Additional loss components, such as sparsity loss on the stimulation protocol, can be implemented to train the network for specific constraints.

The end-to-end model consists of three main components: an encoder, a phosphene simulator, and a decoder (Figure 2). Given an input image, the encoder is designed to find a suitable stimulation protocol, which yields an output map. The value of each element in this stimulation protocol represents the stimulation intensity of one electrode in the stimulation grid. The encoder follows a fully convolutional DNN architecture. In all layers, apart from the output layer, leaky rectified linear units are used as the activation function and batch normalization is used to stabilize training (Table 1). At this time, cortical visual prostheses do not allow for systematic control over phosphene brightness (Najarpour Foroushani et al., 2018; Troyk et al., 2003). Therefore, in this paper, we assume binary instead of graded electrode activation. The Heaviside step function is used as the activation function in the output layer to obtain quantized (binary) stimulation values. A straight-through estimator (Yin et al., 2019) was implemented to maintain the gradient flow during backpropagation.

**Table 1.** Architecture of the encoder component.

	Type	In	Out	Size	Stride	Pad	Normalization	Activation
1	Conv	1	8	3	1	1	BN	LReLU
2	Conv + Pool	8	16	3/2	1	1	BN	LReLU
3	Conv + Pool	16	32	3/2	1	1	BN	LReLU
4	Res	32/32	32/32	3/3	1/1	1/1	BN/BN	LReLU/LReLU
5	Res	32/32	32/32	3/3	1/1	1/1	BN/BN	LReLU/LReLU
6	Res	32/32	32/32	3/3	1/1	1/1	BN/BN	LReLU/LReLU
7	Res	32/32	32/32	3/3	1/1	1/1	BN/BN	LReLU/LReLU
8	Conv	32	16	3	1	1	BN	LReLU
9	Conv	16	1	3	1	1	-	Step

Conv, convolutional layer; Res, residual block; Pool, max-pooling layer; BN, batch normalization; LReLU, leaky rectified linear unit.

In the second component of our model, a phosphene simulator is used to convert the stimulation protocol that is created by the encoder to an SPV representation. This component has no trainable parameters and uses predefined specifications to realistically mimic the perceptual effects of electrical stimulation in visual prosthetics. Phosphene simulation occurs in three steps: first, each element in the 32×32 stimulation protocol is mapped onto prespecified pixels of a 256×256 image, yielding the simulated visual field. Phosphenes are mapped onto a rectangular grid, of which the positions were distorted by a random factor between −0.25 and 0.25 times the phosphene spacing in both horizontal and vertical direction. Second, the phosphene intensities were multiplied with a prespecified random gain value between 0.5 and 1.5 to mimic natural variation in brightness. Third, the obtained image is convolved with a gaussian kernel to simulate the characteristic perceptual effects of electrical point stimulation. In the current experiments, we use a phosphene spacing of eight pixels, and a sigma value of 1.5 pixels for the gaussian kernel.

The third component of our model is the decoder. The decoder is an image-to-image conversion model that is based on a residual network architecture (He, Zhang, Ren, & Sun, 2016), which is known for its useful training properties (Ebrahimi & Abadi, 2018; Huang, Wang, Tao, & Zhao, 2020). Furthermore, residual networks demonstrate computational similarities with recurrent networks that are found in the biological visual system (Kubilius et al., 2019; Liao & Poggio, 2016; Schrimpf et al., 2018). Batch normalization and activation with leaky rectified linear units is implemented in all layers of the model, except for the output layer, which uses sigmoid activation and no batch normalization (Table 2). The decoder component is designed to “interpret” the SPV representation by converting it into a reconstruction of the original input. Our end-to-end architecture implements an auto-encoder architecture, where the SPV representations can be seen as a latent encoding of the original input (or some transformation thereof) (Bengio, Courville, & Vincent, 2013). In this view, the efficient encoding of the rather complex visual environment into a relatively rudimentary phosphene representation can be considered a dimensionality reduction problem in which we aim to maximally preserve the information that is present in the latent SPV representation.

**Table 2.** Architecture of the decoder component.

	Type	In	Out	Size	Stride	Pad	Normalization	Activation
1	Conv	1	16	3	1	1	BN	LReLU
2	Conv	16	32	3	1	1	BN	LReLU
3	Conv	32	64	3	2	1	BN	LReLU
4	Res	64/64	64/64	3/3	1/1	1/1	BN/BN	LReLU/LReLU
5	Res	64/64	64/64	3/3	1/1	1/1	BN/BN	LReLU/LReLU
6	Res	64/64	64/64	3/3	1/1	1/1	BN/BN	LReLU/LReLU
7	Res	64/64	64/64	3/3	1/1	1/1	BN/BN	LReLU/LReLU
8	Conv	64	32	3	1	1	BN	LReLU
9	Conv	32	1	3	1	1	-	Sigmoid

Conv, convolutional layer; Res, residual block; Pool, max-pooling layer; BN, batch normalization; LReLU, leaky rectified linear unit.

## Experiments and results

Model performance was explored via four computational experiments using different datasets. In each experiment, a different combination of loss functions and constraints was tested, as explained below. To quantify reconstruction performance, we report suitable image quality assessment measures. Unless stated otherwise, we report the mean squared error (MSE), the structural similarity index (SSIM; Wang, Bovik, Sheikh, & Simoncelli, 2004), and either the peak signal to noise ratio (PSNR) or the feature similarity index (FSIM; Zhang, Zhang, Mou, & Zhang, 2011) between the reconstruction and the input image, as evaluated using the Scikit-image library (version 0.16.2) for Python (Van Der Walt et al., 2014). Where MSE and PSNR are image quality assessment metrics that operate on pixel intensity, SSIM and FSIM are popular alternatives that better reflect perceptual quality (Preedanan, Kondo, Bunnun, & Kumazawa, 2018). In addition to these performance metrics, we report the average percentage of activated electrodes as a measure for sparsity. Furthermore, to visualize the subjective quality of encodings and reconstructions, we display a subset of images with the corresponding SPV representations and image reconstructions.

### Training procedure

The end-to-end model was implemented in PyTorch version 1.3.1, using a NVIDIA GeForce GTX 1080 TI graphics processing unit (GPU) with CUDA driver version 10.2. The trainable parameters of our model were updated using the Adam optimizer (Kingma & Ba, 2015). The end-to-end model was treated as a single system (i.e. all components of the model were trained simultaneously). To account for potential convergence of the model parameters toward local optima (i.e. to reduce the likelihood that a suitable parameter configuration of the network is missed due to a combination of a specific weights initialization and learning rate), a “random restarts” approach was used. That is, each model was trained five times, each time randomly starting with a different weight initialization. The results only show the best performing one out of these five models (i.e. the one with the lowest loss on the validation dataset), unless stated otherwise.

### Experiment 1: SPV-based reconstruction of visual stimuli

The objective of the first experiment was to test the basic ability of the proposed end-to-end model to encode a stimulus into regular, binary phosphene representations, and decode these into accurate reconstructions of the input image. For this purpose, we trained the model on a self-generated dataset containing basic black and white images with a randomly positioned lowercase alphabetic character. Each image in the dataset is 128×128 pixels, and contains a randomly selected character, displayed in one of 47 fonts (38 fonts for the training dataset and 9 fonts for the validation dataset). The model was trained to minimize the (pixel-wise) MSE.

(1) $$\begin{array}{c} L_{I}=\frac{1}{N}\;\sum_{n=1}^{N}\;{∥x^{\left(n\right)}-{\hat{x}}^{\left(n\right)}∥}^{2}\; \end{array}$$

between the intensities of the input image *x*^(*n*)^ and the output reconstruction ${\hat{x}}^{\left(n\right)}$ over all training examples 1 ≤ *n* ≤ *N*.

**Figure 3.**
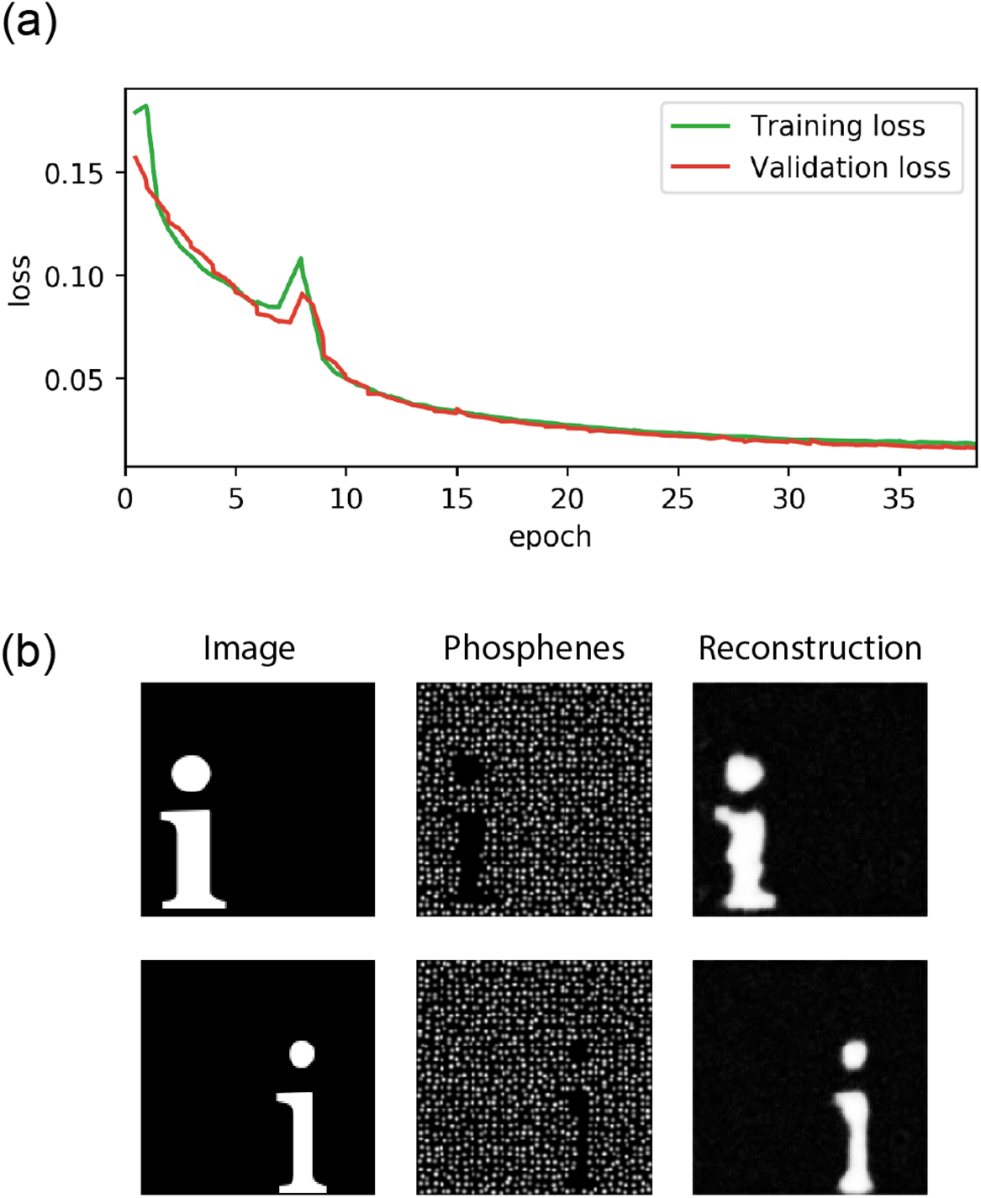
Results of Experiment 1. The model was trained to minimize mean squared error loss. (**a**) The training curves indicating the loss on the training dataset and validation dataset during the training procedure. (**b**) Visualization of the network input (left) the simulated prosthetic vision (middle) and the reconstruction (right).

The results of Experiment 1 for the best performing model out of the five random restarts are displayed in Figure 3. As can be observed, the reconstruction loss is successfully minimized until convergence after 39 epochs. The performance metrics on the validation dataset indicate that the model is capable of adequately reconstructing the input image from the generated SPV representation (MSE = 0.018, SSIM = 0.139, and PSNR = 17.59). Notably, the model adopted an encoding strategy where the presence of the alphabetic character (white pixels) is encoded with the absence of phosphenes and vice versa, resulting in an average electrode activity (percentage of active electrodes) of 95.65%. Such an “inverted” encoding strategy was found for two out of the five random restart initializations and observed for all 234 images in the validation dataset.

### Experiment 2: Shaping phosphene vision via constrained optimization

In the second experiment, we assess whether our model allows the inclusion of additional constraints. To exemplify this advantage of our proposed approach, we chose to evaluate the effects of adding a sparsity loss term. Considering the potentially adverse effects of electrical stimulation (Lewis et al., 2015; McCreery, Agnew, Yuen, & Bullara, 1988), one might want to introduce such a sparsity requirement that constrains the stimulation protocol, limiting the neural degradation by enforcing minimal energy transfer to neural tissue. Let *s* = (*s*_1_,…,*s_M_*) denote a stimulation vector representing the stimulation pattern for *M* electrodes. We define sparsity as the L1 norm on the stimulation protocol:

(2) $$\begin{array}{c} L_{S}=\frac{1}{N}\;\sum_{n=1}^{N}∥s^{\left(n\right)}∥\; \end{array}$$

where *s*^(*n*)^ is the stimulation vector for the *n*-th training example. The objective is to minimize the total loss:

(3) $$\begin{array}{c} L_{total}=\;\left(1-\kappa \right)L_{I}\;+\kappa \;L_{S}\; \end{array}$$

where *L_I_* is the pixel-wise reconstruction loss and the parameter κ can be adjusted to choose the relative weight between the reconstruction loss and sparsity loss. We evaluated 13 values of κ, again each time using the random restarts approach, testing five different weight initializations. We performed a regression analysis on the overall percentage of active electrodes and the reconstruction performance (MSE) to evaluate the effectiveness and the decrease in performance, respectively.

**Figure 4.**
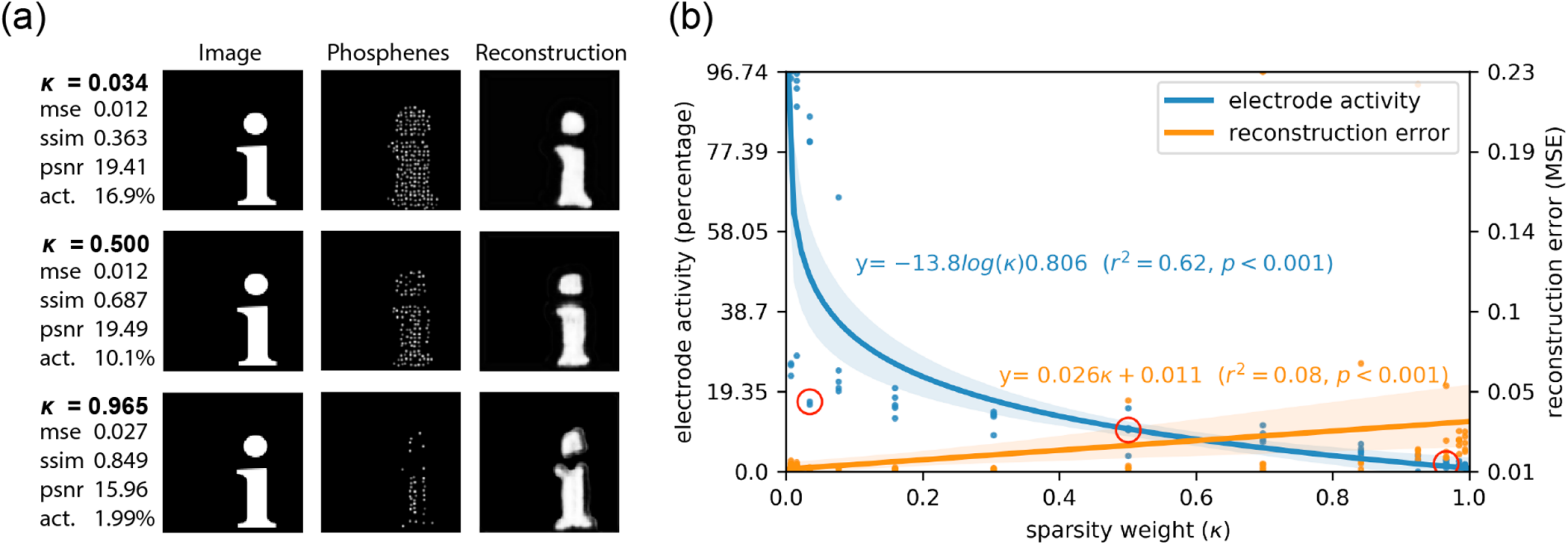
Results of Experiment 2. The model was trained on a combination of mean squared error loss and sparsity loss. The 13 different values for sparsity weight κ were tested. (**a**) Visualization of the results for three out of the 13 values for κ. Each row displays the performance metrics for the best-performing model out of five random restarts, and one input image from, the validation dataset (left), with the corresponding simulated phosphene representation (middle) and reconstruction (right). (**b**) Regression plot displaying the sparsity of electrode activation and the reconstruction error in relation to the sparsity weight κ. The red circles indicate the best-performing model for the corresponding sparsity condition, as visualized in panel **a**.

The results for three of the κ-parameters are displayed in Figure 4. As can be observed, adding additional sparsity loss by increasing the κ parameter resulted in an overall lower percentage of active phosphenes. For larger values of κ, the reconstruction quality dropped. In contrast to the results of Experiment 1, addition of a sparsity loss led to phosphene patterns that more naturally encode the presence of pixels by the presence of phosphenes (instead of vice versa).

### Experiment 3: End-to-end phosphene vision for naturalistic tasks

In the third experiment, the model was trained on a more complex and naturalistic image dataset. To this end, we made use of the ADE20k semantic segmentation dataset (Zhou et al., 2017; Zhou et al., 2019). Compared with the synthetic character dataset, which we used for the aforementioned experiments, one of the key challenges of such naturalistic stimuli is that instead of merely foreground objects on a plain dark background, the images contain abundant information. Here, not all features may be considered relevant, and therefore the task at hand (implemented by a loss function) should control which information needs to be preserved in the phosphene representations. Note that the proposed end-to-end framework allows for optimization to virtually any type of task that can be formalized as a loss function. However, with the current experiments, we merely aimed to demonstrate the basic principle by exploring the encoding strategies for three different types of image reconstruction tasks. We compared the pixel-based MSE reconstruction task that was used in the first experiment with two other types of reconstruction tasks: first, an unsupervised perceptual reconstruction task (see related work by, Johnson, Alahi, and Fei-Fei (2016), Ledig et al. (2017)) which, in contrast to the pixel-wise MSE-based reconstruction task, aims to only preserve high-level perceptual features. Second, a supervised semantic boundary reconstruction task to evaluate the additional value of using labeled supervision to specify which information needs to remain preserved in the reconstructions.

**Figure 5.**
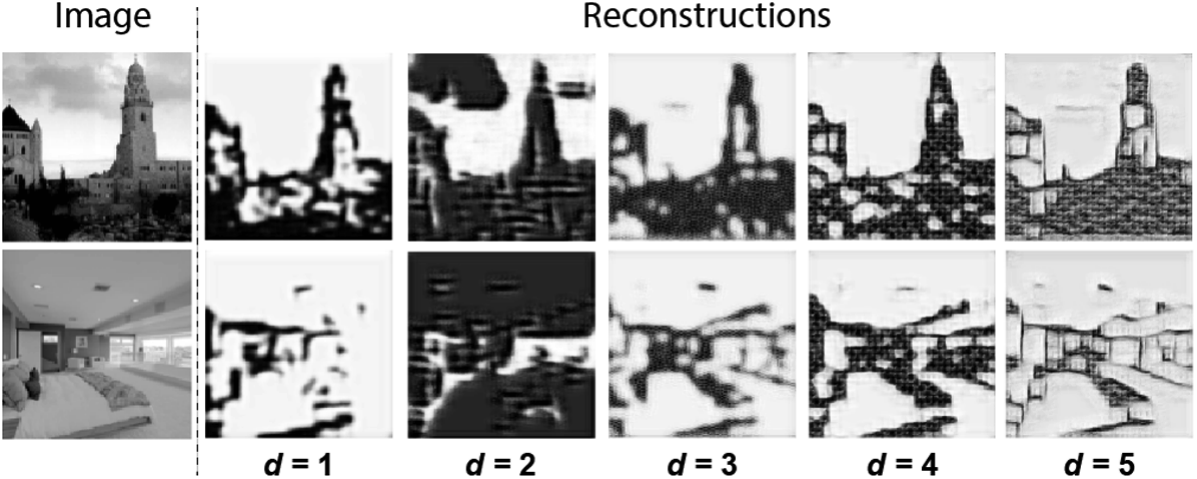
Comparison between different values of d for the perceptual reconstruction task that was used in Experiment 3, where d indicates the layer depth for the VGG-based feature loss.

The perceptual reconstruction task was formulated with the aim of minimizing higher-level perceptual differences between the reconstruction and the input image. These more abstract perceptual differences are defined in feature-space, as opposed to the more explicit per-pixel differences which were used in the previous experiments. The feature loss is defined as

(4) $$\begin{array}{c} \;\;\;\;\;\;\;\;\;\;\;\;L_{P}=\frac{1}{NK}\sum_{n=1}^{N}\;\sum_{k=1}^{K}\;{∥\varphi_{k}^{d}\left(x^{\left(n\right)}\right)-\varphi_{k}^{d}\left({\hat{x}}^{\left(n\right)}\right)∥}^{2}\; \end{array}$$

where N is the number of training examples, $\varphi {\;}^{d}\left(x^{\left(n\right)}\right)$ and $\varphi {\;}^{d}\left({\hat{x}}^{\left(n\right)}\right)\;$are the *d*-th layer feature maps extracted from the input image and the reconstruction image using the VGG16 model pre-trained on the ImageNet dataset (Simonyan & Zisserman, 2015) and *K* is the number of feature maps of that layer. For lower values of *d*, the perceptual loss reveals more explicit differences in low-level features such as intensities and edges, whereas for higher values of *d* the perceptual loss focuses on more abstract textures or conceptual differences between the input and reconstruction (Zeiler & Fergus, 2014). We chose *d* equal to 3 as an optimal depth for the feature loss, based on a comparison between different values (see Figure 5).

In the supervised semantic boundary reconstruction task, the objective was not to minimize the differences between reconstruction and input image. Instead, we aimed to provide labeled supervision that guides the model toward preserving information of semantically defined boundaries. Here, the objective was to minimize the differences between the output prediction of the decoder and processed semantic segmentation target labels of the ADE20K dataset. The semantic segmentation labels that are provided with the dataset were converted to a binary segmentation map representing the boundaries between semantic categories (i.e. boundary pixels have a value of 1 and non-boundary pixels contain the value 0). The reconstruction loss was formalized as the weighted binary cross entropy (BCE), defined as:

(5) $$\begin{array}{ccc} L_{B} & \;= & -\frac{1}{NJ}\sum_{n=1}^{N}\sum_{j=1}^{J}\left(wz_{j}^{\left(n\right)}\log\left({\hat{x}}_{j}^{\left(n\right)}\right)\right.\\ & & \;+\left.\left(1-w\right)\left(1-z_{j}^{\left(n\right)}\right)\log\left(1-{\hat{x}}_{j}^{\left(n\right)}\right)\right)\; \end{array}$$

where $z_{j}^{\left(n\right)}$ is the ground truth boundary segmentation label for pixel *j* of example *n* and *w* is a constant that is introduced for counterbalancing the unequal distribution of non-boundary compared to boundary pixels. In our experiments, w is set equal to 0.925, matching the inverse ratio of boundary pixels to non-boundary pixels. Both for the perceptual loss and the BCE loss we included a sparsity loss as in Experiment 2.

In addition to the three training conditions for our proposed end-to-end model, we included separate conditions where the decoder was trained on SPV representations that were generated using conventional image processing to facilitate a quantitative comparison. These reference SPV images were generated using Canny edge detection (Canny, 1986) and a deep learning-based contour detection method, called holistically nested edge detection (Xie & Tu, 2017). Differences in reconstruction performance were tested for significance using a paired *t*-test on the minibatches of the validation dataset. The significance level α was set to 0.05 and adjusted with a Bonferroni correction to correct for multiple comparisons.

**Figure 6.**
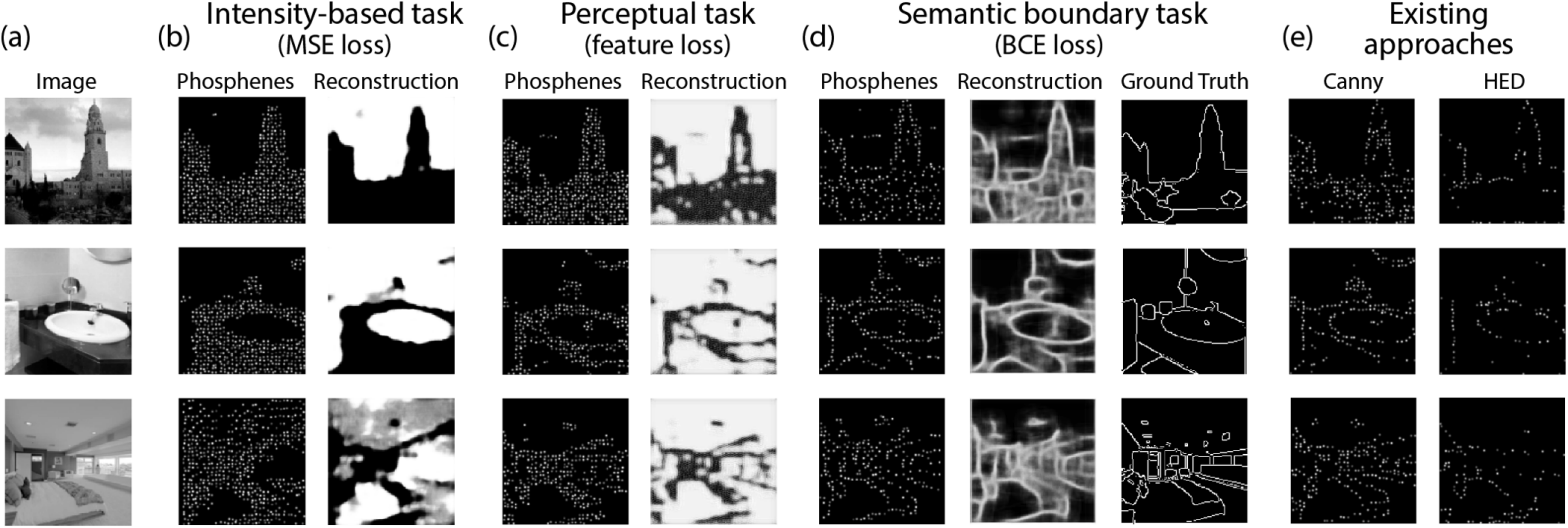
Results of Experiment 3. The model was trained on naturalistic stimuli, comparing three reconstruction tasks. (**a**) Original image. (**b**) Pixel intensity-based reconstruction task with MSE loss (see Equations 1–3). (**c**) Perceptual reconstruction task, using VGG feature loss (see Equation 4; *d* is set equal to 3). (**d**) Semantic boundary reconstruction task, using weighted BCE loss (see Equation 5) between the reconstruction and the ground truth semantic boundary label (i.e. a binary, boundary-based, version of the ground truth label from the dataset). (**e**) Simulated prosthetic percept after conventional image preprocessing with (left) Canny edge detection (Canny, 1986) and (right) holistically nested edge detection (Xie & Tu, 2017).

**Figure 7.**
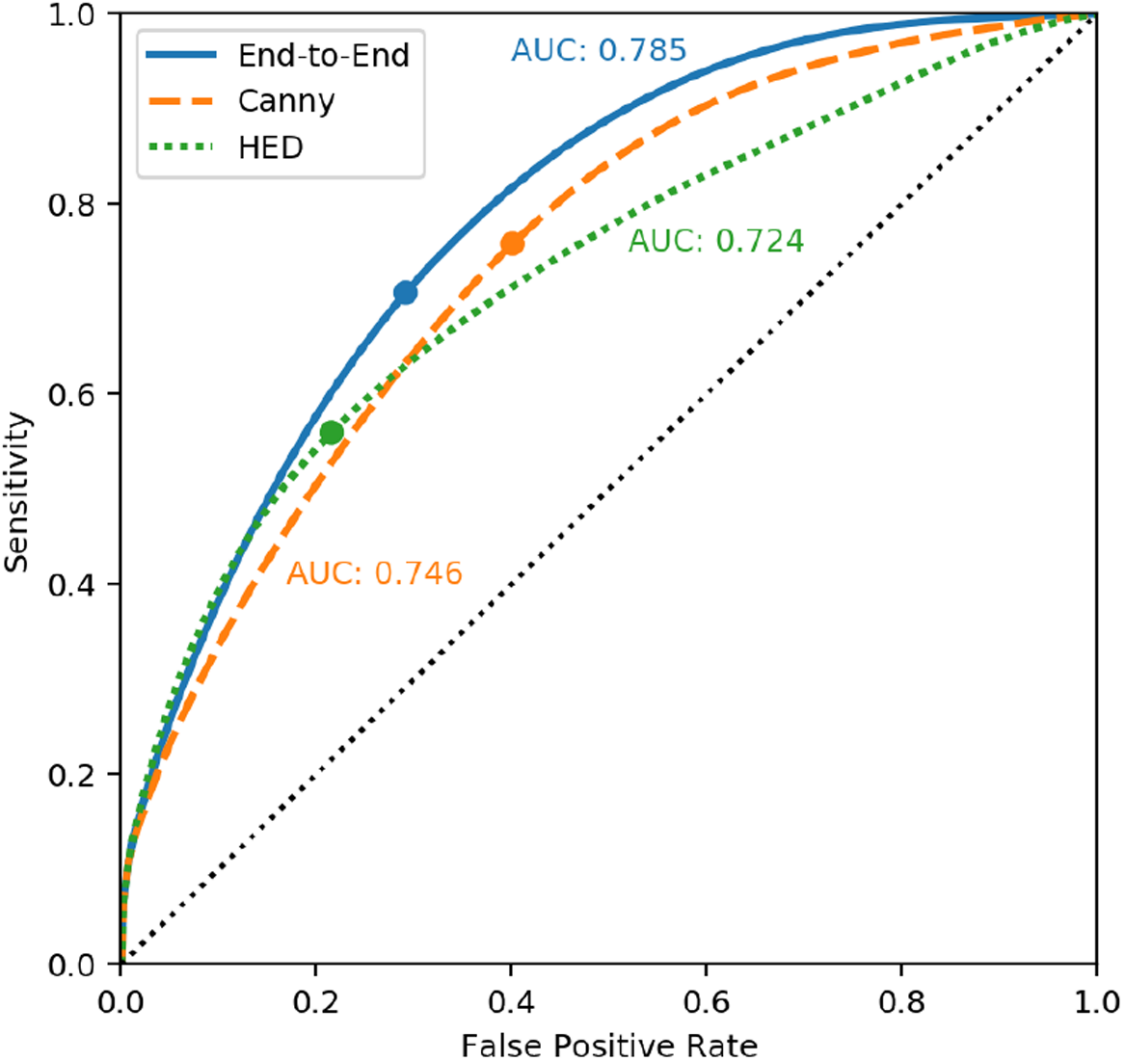
Receiver-operator curves for the semantic boundary prediction task in Experiment 3. Our proposed end-to-end method is compared against existing approaches: Canny edge detection (Canny, 1986) and holistically nested edge detection (Xie & Tu, 2017). The specificity (1 - False Positive Rate), sensitivity and area under the curve (AUC) of the thresholded predictions are also provided in Table 3.

The results of Experiment 3 are displayed in Figure 6, Figure 7, and Table 3. Both for the perceptual reconstruction tasks and the supervised semantic boundary reconstruction task, the model seems to have adopted a different phosphene encoding strategy compared to the intensity-based reconstruction task. The average MSE is significantly lower in the intensity-based reconstruction task, and the average FSIM is significantly higher in the perceptual reconstruction task. In the supervised semantic boundary condition, 69.7% of the boundary pixels were classified correctly.

**Table 3.** Performance metrics for Experiment 3.

	Intensity reconstruction			Perceptual reconstruction			Semantic boundary prediction				
Processing model	MSE	SSIM	FSIM	MSE	SSIM	FSIM	Acc.	Sens.	Spec.	Prec.	AUC
End-to-end (ours)	**0.034**	**0.554**	**0.719**	0.064	**0.541**	**0.761**	0.697	0.722	0.695	0.165	**0.785**
Canny	0.055	0.443	0.672	0.061	0.454	0.581	0.598	**0.774**	0.583	0.134	0.746
HED	0.056	0.454	0.708	**0.059**	0.458	0.588	**0.757**	0.571	**0.772**	**0.173**	0.724

MSE, mean squared error; FSIM, feature similarity index; SSIM, structural similarity index; Acc., accuracy, defined as the proportion of correctly classified pixels; Sens., sensitivity, defined as the proportion of boundary pixels that were correctly identified as such; Spec., specificity, defined as the proportion of non-boundary pixels that were correctly identified as such. AUC, area under the receiver-operator curve.

Significant highest performances are indicated in bold.

Compared to the reconstructions of the SPV-encodings using existing approaches, our end-to-end model achieved adequate reconstruction performance: our model scored the highest performance for the intensity-based reconstruction (MSE, SSIM, and FSIM) and the perceptual reconstruction (SSIM and FSIM). In the semantic boundary reconstruction task, our model scored average for the accuracy, sensitivity, specificity, and precision, and had the largest area under the receiver-operator curve (AUC) compared to the other models.

### Experiment 4: Modular phosphene simulation

Similar to earlier work in the field of simulated prosthetic vision (Bollen, Guclu, et al., 2019; Bollen, van Wezel, et al., 2019; Dagnelie et al., 2007; Parikh et al., 2013; Sanchez-Garcia et al., 2020; Vergnieux et al., 2017; Vergnieux et al., 2014), the previous experiments in this study are performed using a basic simulation of cortical prosthetic vision, with homogeneously distributed, equally-sized phosphenes. In reality, however, the exact phosphene coverage that is achieved in prosthetic vision will depend on many factors, including the electrode placement and the cortical anatomy of the patient, which is variable across people. Both early human trials (Brindley & Lewin, 1968), as well as recent animal studies (Schiller, Slocum, Kwak, Kendall, & Tehovnik, 2011) have shown that individual phosphenes elicited by stimulation in V1 have different sizes, which increase with foveal eccentricity. Srivastava et al. (Srivastava et al., 2007; Srivastava, et al., 2009) developed a more biologically plausible simulation of phosphene vision that explicitly models this effect of cortical magnification for a specific electrode configuration.

In the fourth experiment of our study, we examine an extension of our approach to validate the capacity of our model to optimize for such customized, more realistic, phosphene mappings. Again, we train our end-to-end model, using the ADE20k dataset on the semantic boundary reconstruction task, as described in the previous experiment. However, rather than same-sized phosphenes, placed on a distorted rectangular grid, this time, we use a phosphene map that is inspired by the aforementioned studies of Srivastava et al. (2007, 2009), who simulate phosphenes in the lower left quadrant of the visual field, with phosphene densities and phosphene sizes adjusted in relation to the eccentricity in the visual field to simulate the effect of cortical magnification. For the phosphene simulation, we formalize a custom phosphene map as a set of *n* pre-defined 256×256 greyscale images, {*P*_1_, *P*_2_, …,  *P_n_*}, that each display a single Gaussian-shaped phosphene at a specific location. In our experiment, the number of phosphenes *n* is set to 650, 488, or 325. For each image *P_i_*, we generated a phosphene at polar angle $\phi_{i}∼U\left(\pi ,\frac{3}{2}\pi \right)$, eccentricity $r_{i}=x_{i}+2x_{i}^{2}\;$with $x_{i}∼U\left(0,\;1\right)$ and size σ_*i*_ = 2*r_i_* + 1. After conversion to Cartesian coordinates, *P_i_* covers a square area in the lower left quadrant, bounded by corners (0,   − 1) and (− 1,  0). Note that the described procedure reflects an arbitrary example mapping, which may be replaced to yield any prespecified set of phosphenes. The final SPV image (the output of the simulator) is calculated by taking a weighted sum over all images in the phosphene map:

(6) $$\begin{array}{c} SPV=\sum_{i=1}^{n}w_{i}\;P_{i}\;w_{i}\in \left\{0,\;1\right\}\; \end{array}$$

Here, *w* denotes the stimulation protocol which is the output of the encoder. Note that in order to facilitate the simulation of an arbitrary number of freely distributed phosphenes, the encoder is equipped with a fully connected output layer on top of the architecture which is shown in Table 1. In contrast with convolutional layers, spatial information is lost in a fully connected artificial neural network layer. To preserve spatial coherence between phosphene encodings and the training images, we introduce a regularization term to the cost function, that drives the network to activate phosphenes that correspond with bright regions of the training images and vice versa. This spatial regularization loss is calculated as the BCE loss (see Equation 5) between the output of the encoder and the pixels in the training target, sampled at the location of the phosphene center.

**Figure 8.**
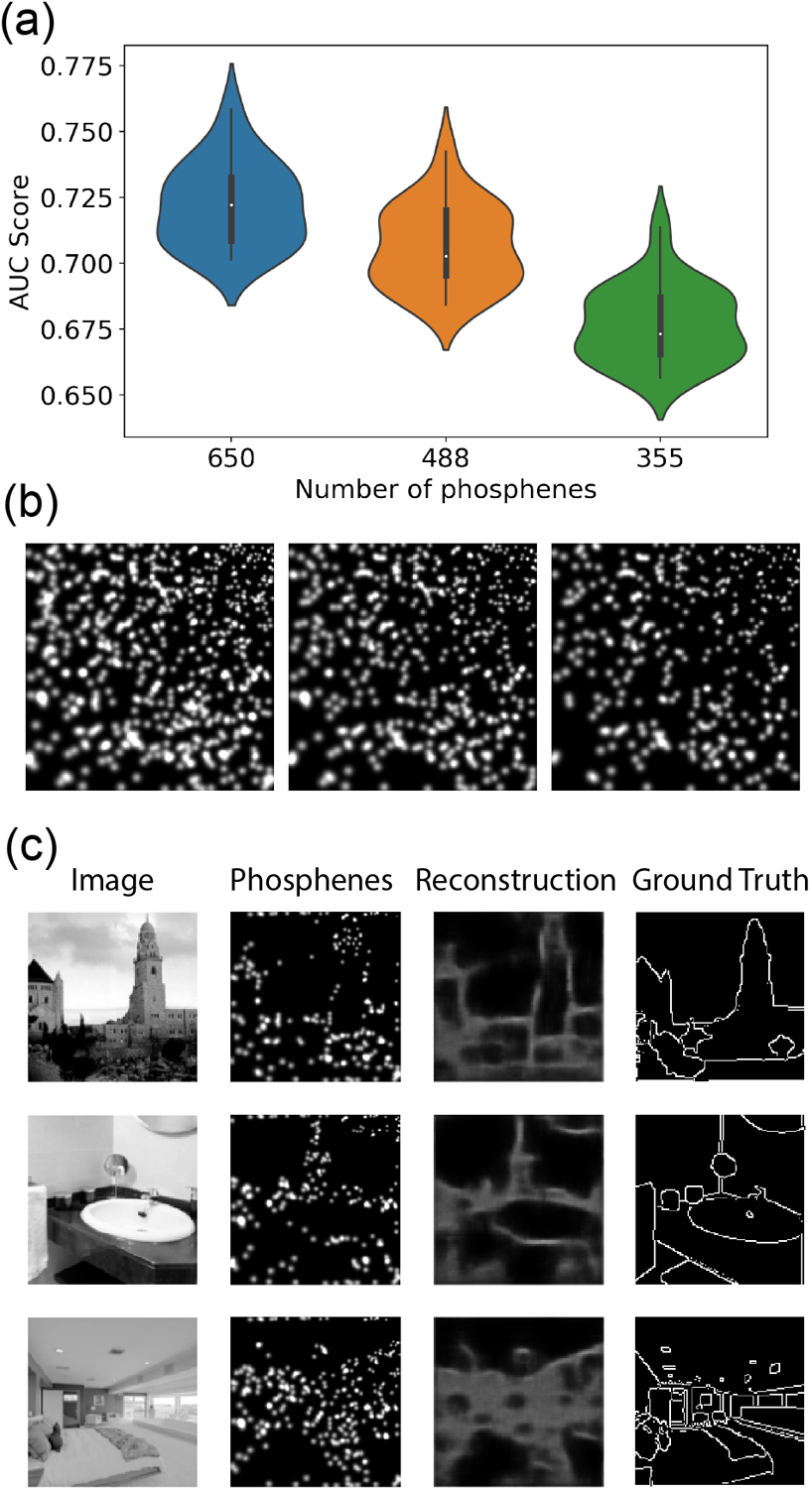
Results of Experiment 4. The model was trained on naturalistic stimuli with a customized phosphene mapping. (**a**) Reconstruction performance for the different phosphene resolutions (AUC: area under the receiver-operator curve). (**b**) Visualization of the phosphene coverage for each resolution (left: 650 phosphenes, middle: 488 phosphenes, and right: 325 phosphenes). (**c**) Validation examples for the training condition with 650 phosphenes.

The model successfully converged to an optimal solution and the results of Experiment 4 are displayed in Figure 8. The reconstruction performance, reflected by the AUC score, was significantly different among the various phosphene resolutions (see Figure 8a). The overall reconstruction performance was lower compared to Experiment 3 (see Figure 7).

## Discussion

In this paper, we present and evaluate a novel deep learning approach for end-to-end optimization of prosthetic vision. Below, we provide a general discussion of the proposed method and the results of our validation experiments, reflecting on the earlier hypothesized automated and tailored optimization abilities. Furthermore, we list some of the limitations of the current study and provide directions for future research.

### Automated optimization

Our end-to-end model is based on an autoencoder architecture, and aims to make use of their well-described ability to efficiently encode information into a low-dimensional latent representation (Bengio et al., 2013). Instead of optimizing image preprocessing as an isolated operation, our approach is designed to automatically optimize the entire process of phosphene generation for a given task. The results from Experiment 1 demonstrate that the model successfully converges to an optimal encoding strategy for a latent representation that consisted of a 32×32 binary simulated phosphene pattern. The model achieved adequate reconstruction performance, as indicated by the low MSE of 0.018 on the validation dataset. Note, that in the unconstrained setting, the model merely maximizes information transfer and has no knowledge about practical requirements, such as sparsity. Therefore, the possible phosphene encoding strategies that may be found by the model are not limited to ecologically useful solutions. This is exemplified by the “inverted” phosphene encoding strategy with a large average electrode activity of 95.65%. Here, finding an undesirable phosphene encoding strategy can be considered an expected consequence of an imprecise learning objective. To guide the model toward useful latent SPV representations, in experiments 2 and 3, we implement additional regularizing constraints (sparsity), and explore different optimization tasks, as discussed below.

An important requirement for automated optimization with deep learning, is that all components of the artificial neural network make use of differentiable operations. In this paper, we contribute a basic implementation of a fully differentiable phosphene simulation module, including a straight-through estimator for quantized phosphene activation. Further studies could adapt or extend this implementation to test automated optimization for different phosphene simulations, for instance, varying the number of electrodes, and the positions of the phosphenes.

### Tailored optimization to sparsity constraints

Implementing additional constraints in the optimization procedure may provide a general solution to account for practical, medical, or biophysical limitations of the prosthetic device. For instance, the inflammatory response of brain tissue is a major concern that limits the long-term viability of cortical electrode implants (Fernández, Alfaro, & González-López, 2020; Polikov, Trresco, & Reichert, 2005) and these adverse effects may partly be avoided by limiting the chronic electrical stimulation itself (Lewis et al., 2015; McCreery et al., 1988). The results of Experiment 2 demonstrate that implementation of such a sparsity constraint may help to regularize the electrode activity. Comparing the results with Experiment 1, we can observe that even with a low value for the sparsity parameter κ, the new objective function causes the model to find a more ecologically useful encoding strategy. Notably, a larger sparsity weight κ results in fewer active electrodes, but also in impaired reconstruction performance. Choosing a balanced value for κ, depending on the needs of the patient, can be seen as a part of a tailored optimization approach of image preprocessing in prosthetic vision.

Importantly, the proposed method enables the implementation of virtually any type of additional constraint that can be incorporated in the optimization procedure. Other examples of biophysical limitations for prosthetic vision, besides sparse electrode activation, could include minimal distance for simultaneously activated electrodes, maximal spread of electrode use, or minimal temporal separation. Future research focusing on such biophysical limitations could extend the proposed method to include such or other constraints in the optimization procedure.

### Task-specific optimization for naturalistic settings

Due to the relative complexity, and the presence of non-relevant information, the encoding of naturalistic scenes into phosphenes remains a challenge and it requires task-dependent processing. This challenge is explicitly addressed by the proposed end-to-end approach.

In Experiment 3, we tested three different reconstruction tasks. The results indicate that for different tasks the model converges to a different optimal encoding strategy, which may indicate task-specific optimization. The higher FSIM and the lower MSE in the perceptual reconstruction task, compared to the intensity-based reconstruction task, indicate that information about the higher-level perceptual features is favored over pixel-intensity information. Similarly, when the model was trained with BCE-loss to reconstruct the processed target labels from the ADE20k dataset, only semantic boundary information was preserved.

The phosphene encodings that were found by the model in this condition are comparable to those found with traditional preprocessing approaches (see Figure 6e), and yield similar reconstruction quality (see Table 3) compared to edge detection (Canny, 1986) or holistic contour detection (Xie & Tu, 2017). Note that a variation on these approaches is investigated in Sanchez-Garcia et al. (2020), who demonstrated that preprocessing with semantic segmentation may successfully improve object recognition performance in simulated prosthetic vision (compared to preprocessing with conventional edge detection techniques). Different from the aforementioned traditional strategies, the proposed end-to-end architecture, merely requires supervision to the output reconstructions and the labels do not directly control the phosphene representations themselves. The proposed end-to-end method takes the advantages of existing deep learning approaches (such as supervision with large precisely labeled data sets) to achieve comparable results. In addition to that, our proposed method provides a generalized approach that opens the possibility for task-specific and tailored optimization.

The VGG feature loss and BCE loss that were implemented in this paper were not chosen only because of their well-established application in optimization problems (Asgari Taghanaki, Abhishek, Cohen, Cohen-Adad, & Hamarneh, 2021; Zhang, Isola, Efros, Shechtman, & Wang, 2018) but also because they represent basic functions that are normally performed in the brain. The feature representations found in deep neural networks illustrate a similar processing hierarchy to that of the visual cortex (Güçlü & van Gerven, 2015; Yamins, Hong, Cadieu, Solomon, Seibert, & DiCarlo, 2014) and boundary detection is one of these processing steps needed for segregation of objects from background (Roelfsema, Lamme, Spekreijse, & Bosch, 2002). Although many details about the downstream information processing of direct stimulation in V1 are yet to be discovered, we know that conscious awareness of a stimulated percept requires coordinated activity across a whole network of brain areas (Bosking, Beauchamp, & Yoshor, 2017). By acting as a digital twin, a well-chosen reconstruction task may mimic the downstream visual processing hierarchy, enabling direct optimization of visual prosthetics to the biological system. Still, fully optimizing the interaction between prosthetic stimulation and the downstream visual processing, requires a deep understanding of the biological networks involved. The proposed end-to-end approach is designed in a modular way and future research can extend the concept with virtually any reconstruction model and task.

### Tailored optimization to realistic phosphene mappings

The precise characteristics of the artificial percept that can be generated with visual prosthetics will depend on many factors, including the electrode placement and the visual cortex of the patient. By including a more realistic simulation module with customized phosphene mapping, we explored the potential of our end-to-end method to optimize for specific phosphene configurations.

The results of Experiment 4 indicate that our end-to-end approach can successfully optimize phosphene encoding for arbitrary configurations. A reduction of the number of available phosphenes from 650 to 488 or 325 phosphenes was associated with reduced reconstruction performance. The overall reconstruction performance with the customized phosphene mapping was lower compared to the regular phosphene mapping that was used in Experiment 3. This reduction may partly be explained by the reduced number of phosphenes. However, this was not formally tested. Possibly, the extension of our model to arbitrary phosphene mappings forms an inherently more challenging task.

Knowledge about the perceived phosphene coverage, which is unique for every patient, can be informative for finding a suitable encoding strategy (Buffoni, Coulombe, & Sawan, 2005; Kiral-Kornek, Savage, O'Sullivan-Greene, Burkitt, & Grayden, 2013). By including a customizable phosphene simulation module in our end-to-end architecture, we aim to provide a tool that can be used for tailored optimization to implant- or patient-specific characteristics.

### Limitations and future directions

Some limitations of the present study provide directions for future research. First, the subjective quality of the phosphene representations is not addressed in the current study. Future research could compare the phosphene encoding strategies found by our proposed model, to existing preprocessing approaches from the current literature, using behavioral experiments. Second, the simulated prosthetic vision that was used in the current study is still a simplified model of the reality, and it does not address several stimulation dynamics, such as pulse frequency, inter-stimulation interval, and interactions between electrodes. Accurate simulation of these characteristics requires further adaptations to our phosphene simulator. In addition, it may be worthwhile to simulate different type of implants. For instance, due to inadvertent activation of underlying axon pathways, phosphenes generated with retinal prostheses may demonstrate distorted shapes that vary across subjects and even individual electrodes (Beyeler et al., 2019). Third, in this paper, the model is trained on static images. Future approaches could extend our end-to-end model to process dynamical stimuli, resembling an even more naturalistic setting and addressing dynamic aspects of the stimulation. Finally, the optimization tasks that were used in the current paper remain basic. Future work could extend the current approach with other or more complex tasks. For instance, with reinforcement learning strategies (see White et al., 2019), the model could be extended to perform tasks that more closely related to the everyday actions that need to be performed by the end-user, such as object manipulation (Levine, Finn, Darrell, & Abbeel, 2015) or object avoidance (LeCun, Muller, Ben, Cosatto, & Flepp, 2005).

## Conclusion

In this paper, we present a novel deep learning-based approach for automated and tailored end-to-end optimization of prosthetic vision. Our validation experiments show that such an approach may help to automatically find a task-specific stimulation protocol, considering an additional sparsity requirement. The presented approach is highly modular and could be extended to dynamically optimize prosthetic vision for everyday tasks and requirements of the end-user.

## References

[bib1] Asgari Taghanaki, S., Abhishek, K., Cohen, J. P., Cohen-Adad, J., & Hamarneh, G. (2021). Deep semantic segmentation of natural and medical images: A review. *Artificial Intelligence Review,* 54(1), 137–178.

[bib2] Beauchamp, M. S., Oswalt, D., Sun, P., Foster, B. L., Magnotti, J. F., Niketeghad, S., et al. (2020). Dynamic stimulation of visual cortex produces form vision in sighted and blind humans. *Cell,* 181(4), 774–783. 32413298 10.1016/j.cell.2020.04.033 PMC7331799

[bib3] Beauchamp, M. S., & Yoshor, D. (2020). Stimulating the brain to restore vision. *Science,* 370(6521), 1168–1169. 33273090 10.1126/science.abf3684

[bib4] Bengio, Y., Courville, A., & Vincent, P. (2013). Representation learning: A review and new perspectives. *IEEE Transactions on Pattern Analysis and Machine Intelligence,* 35(8), 1798–1828. 23787338 10.1109/TPAMI.2013.50

[bib5] Beyeler, M., Nanduri, D., Weiland, J. D., Rokem, A., Boynton, G. M., & Fine, I. (2019). A model of ganglion axon pathways accounts for percepts elicited by retinal implants. *Scientific Reports,* 9(1), 1–16. 31235711 10.1038/s41598-019-45416-4 PMC6591412

[bib6] Bollen, C. J. M., Guclu, U., van Wezel, R. J. A., van Gerven, M. A. J., & Gucluturk, Y. (2019). Simulating neuroprosthetic vision for emotion recognition. *2019 8th International Conference on Affective Computing and Intelligent Interaction Workshops and Demos (ACIIW)*, 85–87.

[bib7] Bollen, C. J. M., van Wezel, R. J. A., van Gerven, M. A. J., & Güçlütürk, Y. (2019). Emotion recognition with simulated phosphene vision. *Proceedings of the 2nd Workshop on Multimedia for Accessible Human Computer Interfaces - MAHCI ’19*, 1–8.

[bib8] Bosking, W. H., Beauchamp, M. S., & Yoshor, D. (2017). Electrical stimulation of visual cortex: Relevance for the development of visual cortical prosthetics. *Annual Review of Vision Science,* 3(1), 141–166. 10.1146/annurev-vision-111815-114525 PMC6916716 28753382

[bib9] Brindley, G. S., & Lewin, W. S. (1968). The sensations produced by electrical stimulation of the visual cortex. *The Journal of Physiology,* 196(2), 479–493. 4871047 10.1113/jphysiol.1968.sp008519 PMC1351724

[bib10] Buffoni, L. X., Coulombe, J., & Sawan, M. (2005). Image processing strategies dedicated to visual cortical stimulators: A survey. *Artificial Organs,* 29(8), 658–664. 16048483 10.1111/j.1525-1594.2005.29104.x

[bib11] Canny, J. (1986). A computational approach to edge detection. *IEEE Transactions on Pattern Analysis and Machine Intelligence,* PAMI-8(6), 679–698. 21869365

[bib12] Chen, X., Wang, F., Fernandez, E., & Roelfsema, P. R. (2020). Shape perception via a high-channel-count neuroprosthesis in monkey visual cortex. *Science,* 370(6521), 1191–1196. 33273097 10.1126/science.abd7435

[bib13] Dagnelie, G., Keane, P., Narla, V., Yang, L., Weiland, J., & Humayun, M. (2007). Real and virtual mobility performance in simulated prosthetic vision. *Journal of Neural Engineering,* 4(1), S92–S101. 17325421 10.1088/1741-2560/4/1/S11

[bib14] Dobelle, W. H., Mladejovsky, M. G., & Girvin, J. P. (1974). Artificial vision for the blind: Electrical stimulation of visual cortex offers hope for a functional prosthesis. *Science,* 183(4123), 440–444. 4808973 10.1126/science.183.4123.440

[bib15] Donti, P. L., Amos, B., & Zico Kolter, J. (2017). Task-based end-to-end model learning in stochastic optimization. *arXiv*, https://arxiv.org/abs/1703.04529.

[bib16] Ebrahimi, M. S., & Abadi, H. K. (2018). Study of residual networks for image recognition. *arXiv*, https://arxiv.org/abs/1805.00325.

[bib17] Fernandez, E. (2018). Development of visual Neuroprostheses: Trends and challenges. *Bioelectronic Medicine,* 4(1), 12. 32232088 10.1186/s42234-018-0013-8 PMC7098238

[bib18] Fernández, E., Alfaro, A., & González-López, P. (2020). Toward long-term communication with the brain in the blind by intracortical stimulation: Challenges and future prospects. *Frontiers in Neuroscience,* 14, 681. 32848535 10.3389/fnins.2020.00681 PMC7431631

[bib19] Güçlü, U., & van Gerven, M. A. J. (2015). Deep neural networks reveal a gradient in the complexity of neural representations across the ventral stream. *Journal of Neuroscience,* 35(27), 10005–10014. 26157000 10.1523/JNEUROSCI.5023-14.2015 PMC6605414

[bib20] He, K., Zhang, X., Ren, S., & Sun, J. (2016). Deep residual learning for image recognition. In *Proceedings of the IEEE Computer Society Conference on Computer Vision and Pattern Recognition* (Vols. 2016 December), 10.1109/CVPR.2016.90

[bib21] Huang, K., Wang, Y., Tao, M., & Zhao, T. (2020). Why do deep residual networks generalize better than deep feedforward networks? – A neural tangent kernel perspective. *Advances in Neural Information Processing Systems,* 33, 2698–2709.

[bib22] Johnson, J., Alahi, A., & Fei-Fei, L. (2016). Perceptual losses for real-time style transfer and super-resolution. *arXiv*, https://arxiv.org/abs/1603.08155.

[bib23] Kingma, D. P., & Ba, J. (2017). Adam: A method for stochastic optimization. *arXiv*, https://arxiv.org/abs/1412.6980.

[bib24] Kiral-Kornek, F. I., Savage, C. O., O'Sullivan-Greene, E., Burkitt, A. N., & Grayden, D. B. (2013). Embracing the irregular: A patient-specific image processing strategy for visual prostheses. *2013 35th Annual International Conference of the IEEE Engineering in Medicine and Biology Society (EMBC)*, 3563–3566, 10.1109/EMBC.2013.6610312 24110499

[bib25] Kubilius, J., Schrimpf, M., Kar, K., Hong, H., Majaj, N. J., Rajalingham, R., et al. (2019). Brain-Like Object Recognition with High-Performing Shallow Recurrent ANNs. *arXiv*, https://arxiv.org/abs/1909.06161.

[bib26] LeCun, Y., Muller, U., Ben, J., Cosatto, E., & Flepp, B. (2005). Off-road obstacle avoidance through end-to-end learning. *Advances in Neural Information Processing Systems*, https://proceedings.neurips.cc/paper/2005.

[bib27] Ledig, C., Theis, L., Huszar, F., Caballero, J., Cunningham, A., Acosta, A., et al. (2017). Photo-realistic single image super-resolution using a generative adversarial network. *2017 IEEE Conference on Computer Vision and Pattern Recognition (CVPR)*, *2017-January* (12), 105–114, 10.1109/CVPR.2017.19

[bib28] Levine, S., Finn, C., Darrell, T., & Abbeel, P. (2015). End-to-end training of deep visuomotor policies. *Journal of Machine Learning Research,* 17, 1–40.

[bib29] Lewis, P. M., Ackland, H. M., Lowery, A. J., & Rosenfeld, J. V. (2015). Restoration of vision in blind individuals using bionic devices: A review with a focus on cortical visual prostheses. *Brain Research,* 1595, 51–73. 25446438 10.1016/j.brainres.2014.11.020

[bib30] Lewis, P. M., Ayton, L. N., Guymer, R. H., Lowery, A. J., Blamey, P. J., Allen, P. J., et al. (2016). Advances in implantable bionic devices for blindness: A review. *ANZ Journal of Surgery,* 86(9), 654–659. 27301783 10.1111/ans.13616 PMC5132139

[bib31] Liao, Q., & Poggio, T. (2016). Bridging the gaps between residual learning, recurrent neural networks and visual cortex. *arXiv*, https://arxiv.org/abs/1604.03640v2.

[bib32] Lozano, A., Suárez, J. S., Soto-Sánchez, C., Garrigós, J., Martínez-Alvarez, J. J., Ferrández, J. M., & Fernández, E. (2020). Neurolight: A deep learning neural interface for cortical visual prostheses. *International Journal of Neural Systems,* 30(9), 1–18. 10.1142/S0129065720500458 32689842

[bib33] McCreery, D. B., Agnew, W. F., Yuen, T. G. H., & Bullara, L. A. (1988). Comparison of neural damage induced by electrical stimulation with faradaic and capacitor electrodes. *Annals of Biomedical Engineering,* 16(5), 463–481. 3189974 10.1007/BF02368010

[bib34] Najarpour Foroushani, A., Pack, C. C., & Sawan, M. (2018). Cortical visual prostheses: From microstimulation to functional percept. *Journal of Neural Engineering,* 15(2), 021005. 29350199 10.1088/1741-2552/aaa904

[bib35] Parikh, N., Itti, L., Humayun, M., & Weiland, J. (2013). Performance of visually guided tasks using simulated prosthetic vision and saliency-based cues. *Journal of Neural Engineering,* 10(2), 026017. 23449023 10.1088/1741-2560/10/2/026017

[bib36] Pezaris, J. S., & Reid, R. C. (2007). Demonstration of artificial visual percepts generated through thalamic microstimulation. *Proceedings of the National Academy of Sciences,* 104(18), 7670–7675. 10.1073/pnas.0608563104 PMC1863473 17452646

[bib37] Polikov, V. S., Tresco, P. A., & Reichert, W. M. (2005). Response of brain tissue to chronically implanted neural electrodes. *Journal of Neuroscience Methods,* 148(1), 1–18. 16198003 10.1016/j.jneumeth.2005.08.015

[bib38] Preedanan, W., Kondo, T., Bunnun, P., & Kumazawa, I. (2018). A comparative study of image quality assessment. *2018 International Workshop on Advanced Image Technology, IWAIT 2018*, 1–4, 10.1109/IWAIT.2018.8369657

[bib39] Riazi-Esfahani, M., Maghami, M., Sodagar, A., Lashay, A., & Riazi-Esfahani, H. (2014). Visual prostheses: The enabling technology to give sight to the blind. *Journal of Ophthalmic and Vision Research,* 9(4), 494. 25709777 10.4103/2008-322X.150830 PMC4329712

[bib40] Roelfsema, P. R., Denys, D., & Klink, P. C. (2018). Mind reading and writing: The future of neurotechnology. *Trends in Cognitive Sciences,* 22(7), 598–610. 29729902 10.1016/j.tics.2018.04.001

[bib41] Roelfsema, P. R., Lamme, V. A. F., Spekreijse, H., & Bosch, H. (2002). Figure - Ground segregation in a recurrent network architecture. *Journal of Cognitive Neuroscience,* 14(4), 525–537. 12126495 10.1162/08989290260045756

[bib42] Sanchez-Garcia, M., Martinez-Cantin, R., & Guerrero, J. J. (2020). Semantic and structural image segmentation for prosthetic vision. *PLoS One,* 15(1), e0227677. 31995568 10.1371/journal.pone.0227677 PMC6988941

[bib43] Schiller, P. H., Slocum, W. M., Kwak, M. C., Kendall, G. L., & Tehovnik, E. J. (2011). New methods devised specify the size and color of the spots monkeys see when striate cortex (area V1) is electrically stimulated. *Proceedings of the National Academy of Sciences of the United States of America,* 108(43), 17809–17814. 21987821 10.1073/pnas.1108337108 PMC3203799

[bib44] Schrimpf, M., Kubilius, J., Hong, H., Majaj, N. J., Rajalingham, R., Issa, E. B., et al. (2018). Brain-score: Which artificial neural network for object recognition is most brain-like? *BioRxiv*, 10.1101/407007

[bib45] Shepherd, R. K., Shivdasani, M. N., Nayagam, D. A. X., Williams, C. E., & Blamey, P. J. (2013). Visual prostheses for the blind. *Trends in Biotechnology,* 31(10), 562–571. 23953722 10.1016/j.tibtech.2013.07.001

[bib46] Simonyan, K., & Zisserman, A. (2015). Very deep convolutional networks for large-scale image recognition. *arXiv*, https://arxiv.org/abs/1409.1556.

[bib47] Srivastava, N. R., Troyk, P. R., & Dagnelie, G. (2009). Detection, eye–hand coordination and virtual mobility performance in simulated vision for a cortical visual prosthesis device. *Journal of Neural Engineering,* 6(3), 035008. 19458397 10.1088/1741-2560/6/3/035008 PMC3902177

[bib48] Srivastava, N. R., Troyk, P. R., Towle, V. L., Curry, D., Schmidt, E., Kufta, C., et al. (2007). Estimating phosphene maps for psychophysical experiments used in testing a cortical visual prosthesis device. *Proceedings of the 3rd International IEEE EMBS Conference on Neural Engineering*, 130–133, 10.1109/CNE.2007.369629.

[bib49] Stevens, G. A., White, R. A., Flaxman, S. R., Price, H., Jonas, J. B., Keeffe, J., et al. (2013). Global prevalence of vision impairment and blindness. *Ophthalmology,* 120(12), 2377–2384. 23850093 10.1016/j.ophtha.2013.05.025

[bib50] Tehovnik, E. J., & Slocum, W. M. (2013). Electrical induction of vision. *Neuroscience & Biobehavioral Reviews,* 37(5), 803–818. 23535445 10.1016/j.neubiorev.2013.03.012

[bib51] Troyk, P., Bak, M., Berg, J., Bradley, D., Cogan, S., Erickson, R., et al. (2003). A model for intracortical visual prosthesis research. *Artificial Organs,* 27(11), 1005–1015. 14616519 10.1046/j.1525-1594.2003.07308.x

[bib52] Van Der Walt, S., Schönberger, J. L., Nunez-Iglesias, J., Boulogne, F., Warner, J. D., Yager, N., et al. (2014). Scikit-image: Image processing in python. *PeerJ,* 2014(1), e453. 10.7717/peerj.453 PMC4081273 25024921

[bib53] Vergnieux, V., Mace, M. J.-M., & Jouffrais, C. (2014). Wayfinding with simulated prosthetic vision: Performance comparison with regular and structure-enhanced renderings. *2014 36th Annual International Conference of the IEEE Engineering in Medicine and Biology Society*, 2585–2588, 10.1109/EMBC.2014.6944151 25570519

[bib54] Vergnieux, V., Macé, M. J. M., & Jouffrais, C. (2017). Simplification of visual rendering in simulated prosthetic vision facilitates navigation. *Artificial Organs,* 41(9), 852–861. 28321887 10.1111/aor.12868

[bib55] Wang, Z., Bovik, A. C., Sheikh, H. R., & Simoncelli, E. P. (2004). Image quality assessment: From error visibility to structural similarity. *IEEE Transactions on Image Processing,* 13(4), 10.1109/TIP.2003.819861 15376593

[bib56] Weiland, J. D., Liu, W., & Humayun, M. S. (2005). Retinal prosthesis. *Annual Review of Biomedical Engineering,* 7, 361–401. 10.1146/annurev.bioeng.7.060804.100435 16004575

[bib57] White, J., Kameneva, T., & McCarthy, C. (2019). Deep reinforcement learning for task-based feature learning in prosthetic vision. *Proceedings of the Annual International Conference of the IEEE Engineering in Medicine and Biology Society, EMBS, 2019*, 2809–2812, 10.1109/EMBC.2019.8856541. 31946477

[bib58] Xie, S., & Tu, Z. (2017). Holistically-nested edge detection. *International Journal of Computer Vision,* 125(1–3), 3–18.

[bib59] Yamins, D. L. K., Hong, H., Cadieu, C. F., Solomon, E. A., Seibert, D., & DiCarlo, J. J. (2014). Performance-optimized hierarchical models predict neural responses in higher visual cortex. *Proceedings of the National Academy of Sciences of the United States of America,* 111(23), 8619–8624. 24812127 10.1073/pnas.1403112111 PMC4060707

[bib60] Yin, P., Lyu, J., Zhang, S., Osher, S., Qi, Y., & Xin, J. (2019). Understanding straight-through estimator in training activation quantized neural nets. *arXiv*, https://arxiv.org/abs/1903.05662.

[bib61] Zeiler, M. D., & Fergus, R. (2014). Visualizing and understanding convolutional networks. *Lecture Notes in Computer Science (Including Subseries Lecture Notes in Artificial Intelligence and Lecture Notes in Bioinformatics)*, *8689 LNCS*(PART 1), 818–833, 10.1007/978-3-319-10590-1_53

[bib62] Zhang, L., Zhang, L., Mou, X., & Zhang, D. (2011). FSIM: A feature similarity index for image quality assessment. *IEEE Transactions on Image Processing,* 20(8), 2378–2386. 21292594 10.1109/TIP.2011.2109730

[bib63] Zhang, R., Isola, P., Efros, A. A., Shechtman, E., & Wang, O. (2018). The unreasonable effectiveness of deep features as a perceptual metric. *arXiv*, https://arxiv.org/abs/1801.03924.

[bib64] Zhou, B., Zhao, H., Puig, X., Fidler, S., Barriuso, A., & Torralba, A. (2017). Scene parsing through ADE20K dataset. In *Proceedings - 30th IEEE Conference on Computer Vision and Pattern Recognition, CVPR 2017* (Vols. 2017-January), 10.1109/CVPR.2017.544

[bib65] Zhou, B., Zhao, H., Puig, X., Xiao, T., Fidler, S., Barriuso, A., & Torralba, A. (2019). Semantic understanding of scenes through the ADE20K dataset. *International Journal of Computer Vision,* 127(3), 302–321.

[bib66] Zrenner, E., Bartz-Schmidt, K. U., Benav, H., Besch, D., Bruckmann, A., Gabel, V.-P., Gekeler, F., et al. (2011). Subretinal electronic chips allow blind patients to read letters and combine them to words. *Proceedings of the Royal Society B: Biological Sciences,* 278(1711), 1489–1497. 10.1098/rspb.2010.1747 PMC3081743 21047851

